# Supplementary material for: SLAM-based augmented reality for the assessment of short-term spatial memory. A comparative study of visual versus tactile stimuli
Source: PLoS One. 2021 Feb 4;16(2):e0245976. doi: 10.1371/journal.pone.0245976 (PMC7861452; doi:10.1371/journal.pone.0245976)
Supplement: S1 Appendix — (DOCX) [file pone.0245976.s001.docx]

**Appendix**

**Questionnaire for the subjective experience**

| **QUESTION** | **VARIABLE** |
| --- | --- |
| 1. I really enjoyed doing this activity. | enjoyment |
| 1. I have focused on the tasks that I had to do and not on the control mechanisms. | concentration |
| 1. The application was easy to handle. | usability |
| 1. It was easy to learn how to use the application. | usability |
| 1. I do not need the help of an expert to use this application. | usability |
| 1. I am satisfied with how I have done it. | competence |
| 1. I was calm during the experience. | calmness |
| 1. At the end of the experience I felt like an expert in handling the application. | expertise |
| 1. The use of the application did not require great mental effort. | non-mental effort |
| 1. The use of the mobile phone did not require great effort by arms or hands. | non-physical effort |
| 1. I would like to use the application again. | satisfaction |
| 1. I would like to use this technology for other uses. | satisfaction |
| 1. I was able to examine the geometrical shapes closely. | presence |
| 1. I was able to examine the geometrical shapes from different viewpoints. | presence |
| 1. There were moments during the experience when I thought the geometrical shapes were real. | presence |
| 1. When you reflect and think about the experience you have had, you remember the geometrical shapes as objects that were in the room. | presence |
| 1. I had the feeling that the geometrical shapes were in the room. | presence |
| 1. I did not pay attention to the difference between the geometrical shapes and the real world. | presence |
| 1. The control mechanisms did not distract me. | concentration |
| 1. I liked how the geometric shapes looked. | satisfaction |
| 1. Rate the experience [1-7] | satisfaction |
