# Supplementary material for: SLAM-based augmented reality for the assessment of short-term spatial memory. A comparative study of visual versus tactile stimuli
Source: PLoS One. 2021 Feb 4;16(2):e0245976. doi: 10.1371/journal.pone.0245976 (PMC7861452; doi:10.1371/journal.pone.0245976)
Supplement: Attachment — Submitted filename: Response to Reviewers.pdf [file pone.0245976.s003.pdf]

PONE-D-20-20120
A SLAM-based augmented reality framework for the assessment of short-term spatial memory. A comparative study of visual versus tactile stimuli
PLOS ONE

We thank the editor and the reviewer for their valuable comments and suggestions. We have modified the paper according to these comments and suggestions. We hope, the improvements made to the paper make it suitable for publication in PLOS ONE. We have recognized the contribution of the editor and the reviewer in the acknowledgment section. A native English speaker has revised the English of the text included in the paper. This expert has not reviewed our answers in this document.

Following the editor's guidelines for incorporating changes in the manuscript, the added or revised text is marked with a yellow background. Removed text is crossed out and use a red font. However, to improve readability, we have not marked all the removed text, especially in the state of the art, but we have indicated this fact in the comments below.

In the following pages, we address the comments of editor/reviewer (in black). Our answers to these comments use a dark blue font.

Editor comments:
1) Sections 1.1 and 2.2 in the Introduction are overly lengthy. All of the literature discussed is indeed relevant and should be mentioned. However, I suggest the authors summarize the main findings of the studies they mention, reduce this whole section to a couple of paragraphs, and move most of the content of these two sections to the Discussion section of the manuscript. Even for the portions of these section that will be moved to the Discussion, I would strongly suggest that many details should be removed altogether. For example, there is no need to specify the number of participants in each study, or the details of experimental conditions. The authors should simply summarize in one sentence the findings of each study and comment on how their current work relates to this previous literature.
We understand the editor refers to sections 2.1 and 2.2.
We have reduced these two sections. We have left one paragraph for VR, one paragraph for AR works related to spatial memory and a last paragraph for other AR works not as closely related. To facilitate reading, we have not marked the removed text. We have removed the second level headings. We have moved one complete paragraph to the Discussion section.

2) Page 19 line 513: "This guaranteed that the participants could not remember the location of the shapes." This claim is unsubstantiated. It is well possible that participants at least partly remembered the location of objects from one session to the next. The fact that the vision session always occurred after the haptic session is thus a confound. This means that we cannot completely exclude that the better performance in the vision session was due to participants remembering shape locations from the previous session. Ideally, the study should be repeated with new participants, and by randomizing object location and counterbalancing session order. I strongly suggest the authors adopt these practices in future studies. For the current study, I imagine collecting new data would be challenging due to the covid-19 pandemic. Therefore, at the very least this limitation of the study should be clearly noted in the discussion section of the manuscript.
We have changed "This guaranteed that the participants could not remember the location of the shapes" by "After more than two months, it was very difficult for the participants to remember the location of the shapes". We have not mentioned it in the paper, but those

two months were the typical vacation months in Spain. This circumstance, the time passed and the type of objects used, incline us to think that the ability to recall the location of each shape, if any, would be minimal. However, as the editor argues, we cannot guarantee it 100%.
We appreciate the editor's advice. Although we would like to add that in most of the studies, we counterbalance the participants, the ages, the type of session, etc. However, in this specific study, although we had everything prepared to do it, we needed several rooms to be able to carry out the two sessions alternatively. We did not have so many different rooms to be able to carry them out simultaneously. We have added this limitation in the discussion section as follows:
"A limitation of our study is that the two sessions were carried out in the same order (first the tactile and then the visual). The design of our study could have influenced the better performance in the vision session due to participants remembering shape locations from the previous session. Nevertheless, we would like to highlight that the participants carried out the second session more than two months later, the type of objects used were not remarkable to induce a long-term memory, and the room in which the study was carried out was not familiar to the participants. These three aspects lead us to argue that it would be very difficult for a participant to remember the specific location of a certain geometric shape in the room. However, this limitation could have been removed if the order of the sessions were counterbalanced."

3) Results need to be presented more clearly and rigorously.
a. To begin with, it would be useful to have plots showing the differences between haptic and vision sessions for each of the performance outcomes. Presenting medians and IQRs in parentheses scattered throughout the results makes it very difficult to understand and interpret the findings.
We have added four plots for the performance outcomes variables related to the use of the application for the two conditions.

b. Asterisk notations should be kept consistent across the manuscript. In most places ** means $p<0.05$, but in Table 1 *$p < .05$ and ** $p< .01$. I would suggest removing asterisks from the main test, and only showing them in figures and tables, employing them in the standard notation: *$p < .05$; ** $p< .01$; ** $p< .001$
We have removed ** from the main text. We have not marked the removals in the text. We have replaced "The symbol ** indicates a statistically significant difference at level $\alpha = .05$" with "The results were considered to be statistically significant if $p < .05$."

c. Page 23 line 622. This sentence is unclear and I do not understand what correlations were actually assessed.
We have clarified this sentence detailing the variables as follows
"We also used the Spearman rank correlation to test the associations among the ten subjective variables (enjoyment, concentration, etc.) and the variables of the performance outcomes (LocShapes and AttemptS)."

d. Even though I do not fully understand exactly which correlations were tested, I believe the authors must have performed at least 60 correlation tests. Some correction for multiple comparisons needs to be employed. All non-significant results should not be discussed. For example, the authors should remove from page 22 line 601, and from the abstract, the mention of a "tendency" for gender difference. The authors should also remove lines 609 to 612, since the results for men and women appear to be the same.
The following reorganization has not been suggested, but we have rearranged the Correlation subsection for clarity. We have moved the Correlations subsection (Previous

5.3) after the previous 5.4 Subjective perception in order to include all of the correlations in the same section. In the original paper, the correlations were discussed in section 5.3 and in section 5.4.
We have applied the Bonferroni correction to the calculated correlations. We have marked the changes in the Correlations subsection. We have not marked the removed text from previous calculations. The Abstract and the Discussion have also been modified to clarify this point.
We have removed the following sentences:

- ", a tendency for women requiring fewer attempts than men for the tactile condition was found" (abstract).
- "However, we did find a tendency of women requiring fewer attempts than men for TactileCondition (p = .063)" (previous lines 601-602).
- "For the Tactile Condition, no significant correlations were found between the AR spatial task and other tasks" (previous lines 612-613).

We have also removed the following sentences:

- "For women, there are no significant correlations between LocShapes and the subjective variables" (Subsection: 5.4. Subjective perception).
- "For the women, there are no significant correlations between this time variable and the subjective variables" (Subsection 5.4. Subjective perception).

e. It would also be useful to see scatter plots for all of the significant correlations the authors describe.
Figure 7 shows all of the scatter plots for the significant correlations after applying the Bonferroni correction.

4) All of the data underlying the results of the manuscript needs to be made available. I would suggest the authors upload all their data and relevant analysis code to a public data repository such as Zenodo. If the authors do not wish to upload their data to a public repository before the manuscript is accepted, they should at least upload this data as supplementary material, or provide a private link where I can assess whether all the data is presented in a format that allows other researchers to reproduce the results presented in the study. It would also be useful and good research practice if the authors uploaded the software they describe in the manuscript (i.e. the various "apps") to a public repository as well.
We attach the DB in a Supplementary File. This DB can be published as a supplementary file associated with the paper.

Page 2 Line 39: "Most of the information that humans explicitly store in spatial memory comes from the visual and auditory modalities." Please substantiate this claim with appropriate references.
We have added 4 references to substantiate this sentence. We have also added "From neurobiological and cognitive perspectives" at the beginning of the sentence. The sentence now is as follows:
"From neurobiological and cognitive perspectives, most of the information that humans explicitly store in spatial memory comes from the visual and auditory modalities [2-5]."

Page 2 Line 55: "In contrast, physical displacement has been shown to be important in spatial ability" This sentence is unclear, I do not understand the point the authors are trying to make. Please rephrase/elaborate.
We think the problem is "In contrast" that should be "Moreover". Both sentences are to highlight the importance of physical displacement for spatial ability in real life. We have

changed “In contrast” by “Moreover”. We have also modified the sentence as follows: “Moreover, physical displacement has been shown to be important in acquiring spatial ability skills”

Page 11 line 293: “PTSOT” please define this acronym the first time it appears in the text.
We have defined this acronym.

Page 16: Please specify the tasks of the Shape discrimination and Spatial location tests. What were the participants reporting?
To clarify the protocol, we have added in the first test (Spatial orientation test) the following “… the participants must indicate verbally whether or not … The supervisor writes down their answers without giving any feedback.”
In the Shape discrimination test, we have added “As in the previous test, the participants must inspect the reference element by touch. Then, for each of the four elements, the participants must indicate verbally whether or not the shape of the figure is the same as the reference element”
In the Spatial location test, we have added “As in previous tests, the participants must inspect the reference element by touch. Then, for each of the four elements, the participants must indicate verbally whether or not the spatial location of the inner elements is the same spatial location as in the reference element.”

Page 17: the meaning of the numbers in parentheses - e.g. line 446: “(28; 3.5)” - is unclear, since this is explained only on page 20.
Thank you. We have moved the details about the statistical tests from the introduction of Results to a new subsection (4.2. Statistical tests), before the Inclusion criteria subsection (now 4.3). We have modified the explanation about the descriptor of each group as follows: “A descriptor of each group is presented in the format (median (Mdn); interquartile range (IQR)).” For clarity we have changed values such as “(28; 3.5)” by “(Mdn = 28; IQR = 3.5)”. The rest of the numbers in parentheses in this paragraph have also been changed. We have also used the same format for the numbers in parentheses that appear in the Results section. We have not marked all of these changes in the text.

Page 17 line 473: “One men” should be “One man”, or “One male”
We have corrected it.

Page 22 line 608 and line 624: “p > ” should be “p <”
Thank you. We have corrected the two ‘p < ‘.

Page 25 line 678 “On a scale from 1 to 7, the medians were very high: equal to or above 6 in all cases (except for one with a value of 5.3).” This statement is unclear. The reader does not know what the questions on the questionnaire were, so one cannot judge what “high medians” represent.
We have clarified the scale used in the questionnaire when it is described in Section 4.4 Measures. We have also added the questionnaire in a Supporting Information file (S1_Appendix).

Journal Requirements:
1. Please ensure that your manuscript meets PLOS ONE's style requirements, including those for file naming. The PLOS ONE style templates can be found at https://journals.plos.org/plosone/s/file?id=wjVg/PLOSOne_formatting_sample_main_body.pdf and https://journals.plos.org/plosone/s/file?id=ba62/PLOSOne_formatting_sample_title_authors_affiliations.pdf
We have revised the journal requirements and have modified the size of Level 1-2 Headings, the citation of Figures and the style for the references (PLOS ONE in Mendeley).

2. We note that Figure 2 includes an image of a participant in the study.
As per the PLOS ONE policy (http://journals.plos.org/plosone/s/submission-guidelines#loc-human-subjects-research) on papers that include identifying, or potentially identifying, information, the individual(s) or parent(s)/guardian(s) must be informed of the terms of the PLOS open-access (CC-BY) license and provide specific permission for publication of these details under the terms of this license. Please download the Consent Form for Publication in a PLOS Journal (http://journals.plos.org/plosone/s/file?id=8ce6/plos-consent-form-english.pdf). The signed consent form should not be submitted with the manuscript, but should be securely filed in the individual's case notes. Please amend the methods section and ethics statement of the manuscript to explicitly state that the patient/participant has provided consent for publication: "The individual in this manuscript has given written informed consent (as outlined in PLOS consent form) to publish these case details".
If you are unable to obtain consent from the subject of the photograph, you will need to remove the figure and any other textual identifying information or case descriptions for this individual.
The subject of the photograph that appears in Figure 2 is the first author of the paper. He gave written informed consent (as outlined in PLOS consent form) to publish these case details. We have added the image of another user. He also gave written informed consent to publish these case details.
We have also amended the ethics statement of the manuscript as follow "The individuals who appear in the images of this manuscript gave written informed consent to publish their case details".

3. We note that you have stated that you will provide repository information for your data at acceptance. Should your manuscript be accepted for publication, we will hold it until you provide the relevant accession numbers or DOIs necessary to access your data. If you wish to make changes to your Data Availability statement, please describe these changes in your cover letter and we will update your Data Availability statement to reflect the information you provide.
We include the data in a supporting information file (S2 DB). We changed the Data Availability statement as follows "All relevant data will be within the manuscript and its Supporting Information files".

4. Your ethics statement should only appear in the Methods section of your manuscript. If your ethics statement is written in any section besides the Methods, please move it to the Methods section and delete it from any other section. Please ensure that your ethics statement is included in your manuscript, as the ethics statement entered into the online submission form will not be published alongside your manuscript.
The ethics statement only appears in the Study section

Reviewer #1

The paper describes an AR framework for the assessment of short-term spatial memory. The presented work has a good basis of prior work, already published by the same authors. The differences and the novelty of the present work have been outlined and described by the authors. Nevertheless, I am not sure that PLOS one is the right journal for a work that I would define as "incremental". Without considering this issue, I think that the work is valuable, interesting, and supported by good results. Results seems complete and well presented.

Thank you

1) Language. In some points authors used a sort of colloquial language. As an example, in the abstract they wrote "This paper presents a framework for the development of SLAM-based Augmented Reality apps for the assessment of spatial memory". What is the meaning of apps? Have you developed a framework (as in the title), a software, a mobile application? You should better define the software contribution of the work.

We have changed all ‘app’ appearances by ‘application’.

We have developed a framework for SLAM-based AR applications, and using this framework we developed the AR application for the study presented in this paper. The framework has not been previously described in any of our previous publications. The AR app developed for this study with the framework has not been previously presented in any of our previous publications. We have added the following sentence in the Abstract to clarify this aspect “An AR mobile application was developed for this type of assessment involving visual and tactile stimuli by using our framework.”

We have also modified the related sentences in the introduction section as follows:

“This work presents a framework for the development of AR based on SLAM (Simultaneous Localization and Mapping). An AR mobile application was developed for this type of assessment involving visual and tactile stimuli by using our framework. Our AR application can be used …”

We have also changed the title as: “SLAM-based augmented reality for the assessment of short-term spatial memory. A comparative study of visual versus tactile stimuli”

2) The previous point also has a consequence in the rest of the paper. In general, it is difficult for the reader to understand the real contribution of the paper. You described the framework/software/app but it is difficult for the reader to understand how it works. You should add more images showing the app working in the different cases, and better some link to videos showing the app working in the different modalities. You should better explain how the app works in the tactile situation.

We have homogenized the terms used throughout the paper.

We have included two new figures (Fig 2 and 4) that graphically show the steps followed by the users in the two conditions. We have improved the description of the steps followed by users when participating in the tactile condition (Section 3.3).

3) Again about the choice of the terms, I am not convinced about the name "visual app", to define the AR software that shows virtual object in AR. As far as I understood the "visual app" is a "standard" AR app based on SLAM, so a less confusing name should be used to refer to it.

Thank you. We have changed “visual app” by “AR application”. We have also clarified the introduction of section 3. We have divided section 3.1 in two. The headings of these sections have also been changed.

4) When describing the software, the same level of details has been provided for new features and for already implemented features (like the scanning if the room). You should better explain what is from the standard SDK you used and what you have developed.
To clarify this point, we have changed the introduction of Section 3.7. We have also updated Fig. 6 including how external AR SDKs are integrated in our framework.

5) Section 4 “Study” should be carefully checked. There are repetitions (see “The gender distribution was 70% men.} that is repeated twice), and numbers are given in a non-uniform way.
The first “The gender distribution was 70% men” refers to the initial sample (53 adults). The second “The gender distribution was 70% men” refers to the final sample (47 adults). The percentages are the same, but they could be different.
With regard to the mean and standard deviation of age, we have changed the two related sentences as follows:
“The mean (standard deviation) age of the participants was 30.18 (9.25) for males, and 32.86 (11.22) for females.”

6) Questionnaires: since you used both standard questions and ad-hoc ones, you should provide the list of questions as they have been used at least in an appendix or as an external link.
Thank you. We have added the questionnaire in a Supporting Information file (S1_Appendix).

7) Discussion. The works strongly rely on Tango SDK, which has been deprecated. You discussed it, but it is a big issue for the further development and use of the system you developed.
We understand the reviewer's concern. However, our framework integrates Tango SDK, but it can use different AR engines/SDKs such as ARCore or ARKit. As argued in the Discussion section, our framework can develop applications that are independent of the specific AR SDKs. The Tango SDK can be replaced with an equivalent engine without altering the application code. In fact, we are incorporating ARKit in our framework to migrate our app to the iPad Pro.

7b) Discussion: there is a big gap between discussion of sw related issues and discussion about other aspects, like the memory impairments (see lines 699-700).
Thank you. We have added two sentences before the discussion about memory impairments. These sentences are as follows:
“Our proposal and other similar tools can greatly help in training short-term spatial memory. Difficulties in spatial memory are usually associated to disorders or diseases (e.g., [47][48]). For example, several studies have reported…”
