# Supplementary material for: SLAM-based augmented reality for the assessment of short-term spatial memory. A comparative study of visual versus tactile stimuli
Source: PLoS One. 2021 Feb 4;16(2):e0245976. doi: 10.1371/journal.pone.0245976 (PMC7861452; doi:10.1371/journal.pone.0245976)
Supplement: Attachment — Submitted filename: Response to Reviewers_R2.pdf [file pone.0245976.s004.pdf]

PONE-D-20-20120R2
SLAM-based augmented reality for the assessment of short-term spatial memory. A comparative study of visual versus tactile stimuli
PLOS ONE

We greatly appreciate the editor and the reviewer for their help in improving the manuscript. We have modified the manuscript according to your comments and suggestions. We hope, the improvements made to the manuscript make it suitable for publication in PLOS ONE. As in the first review, a native English speaker has revised the English of the text included in the manuscript. This expert has not reviewed the contents of this document.

Following the editor's guidelines for incorporating changes in the manuscript, the added or revised text is marked with a yellow background. Removed text is crossed out and colored in red.

In the following pages, we address the comments of editor/reviewer (in black). Our answers to these comments are in blue.

General comment:
Both Reviewer 1 and myself were pleased with how you addressed all the comments we raised. I have only a few remaining minor changes I would ask you to make, again to aid the clarity of the presented figures and results. Note that Reviewer 1 also commented on the quality of the figure resolution, so I recommend you double check the PlosOne figure formatting guidelines and make sure you upload all figure files at the right size, resolution, and with visible text. The remaining edits to the manuscript should not take much effort, and I don't think there will be need for further rounds of revisions after this. I look forward to receiving your revised manuscript.
Thank you. Figures 8 and 9 have been generated again following your recommendations. We have checked that these figures have a resolution of 300 dpis, a width of about 11 cm, with 1384 pixels. We attach the figures in separated tif files. We also double checked the PlosOne figure formatting guidelines. We hope, now, the figures are suitable for publication.

Editor Comments:
There is a sentence typo on line 117: "have recently presented a development and study that…"
Thank you. We have added 'a' before study.

Section 5.3 should probably not be a section on its own, but can be incorporated in the previous section 5.2, since you are still evaluating gender effects.
We have joined the two sections and modified the title to reflect the content.

In Section 5.4, it would be useful if you explained in simple terms what the reported correlations mean. For example, you say: “LocShapes correlates with MapShapes (r = .49, p < .01), and AttemptS also correlates with MapShapes (r = -.48, p < .01).”. You could elaborate specifying that: “This sensibly means that participants who placed fewer shapes correctly during the evaluation phase or required more attempts to place the shapes correctly also made more mistakes on the map pointing task.” I know you elaborate on these results in the discussion section, but it’s difficult to keep in mind what all the variables here mean. Thus very simple explanations would be useful for all the correlations reported.
Thank you very much indeed. We have added your suggested sentence and also other sentences for simple explanations about the rest of correlations.

I appreciate the new result figures; they greatly aid comprehension. These could still be improved in a few minor ways.
Figures 8 and 9 have been generated again following your recommendations.

- Text size should be increased to improve readability.
We have increased the text size of Figures 8 and 9. We have checked that these figures have a resolution of 300 dpis, a width of about 11 cm, with 1384 pixels.

- In all panels of figures 8 and 9 I suggest you scale the axes so they cover the full range of possible values starting from 0.
We have scaled the axes starting from 0 in Figure 8. We have not done the same in Figure 9 because more than half of the space of the plot area would be empty. Next, we include an example. In any case, if you prefer all panels of Figure 9 with the axes starting from 0, let us know and we will change it.

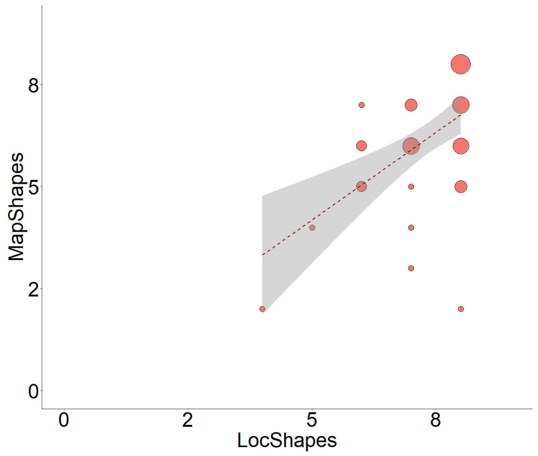

- Where appropriate, please specify axes units, for example in Figure 8b, the y axis label could be “TTimeL (s) ”.
We have added axes units when time variables are used in Figures 8 and 9.

- To reduce clutter, only one legend is sufficient in Figure 8, since all subpanels have the same color scheme.
We have removed all the legends altogether, since the axes are labelled.

- In Figure 9, you could make all the axes square. More importantly, some dots in the scatterplots actually represent multiple data points. To show this, you could scale dot size by the number of occurrences for each data point. For an example see Figure 4 from https://doi.org/10.1080/07370024.2016.1243478

Thank you. In Figure 9, we have left the axes square. The plots in Figure 9 have been generated again following your recommendations. We have used the occurrences of the same pair of values represented in the previous version by a single point and we have represented them by three different circle sizes. These three sizes of circles represent a low number of occurrences, an intermediate number of occurrences, and a high number of occurrences. We have considered doing this categorization and not scaling the circles based on exact occurrences, because there is quite a difference among the occurrences of the different plots. In addition, drawing the size of the circles as a function of their exact occurrences made the information hard to understand. We had to set a minimum radius for small occurrences, since occurrences of 1 were dots and were unnoticeable. In addition, the plots have been generated also including a maximum radius, so that the size of the circles are similar in all the plots.

In the results, it looks like you run correlation analyses only for the visual condition (lines 638-639). However, in the discussion section (line 718) you say that “In contrast, spatial-tactile associations were learned, but failed to achieve this transfer.” Does this mean you performed the same correlation analyses for the tactile condition and found no significant results? If so, please specify this. This probably means you simply have to add the previously-deleted sentence in the results: “For the Tactile Condition, no significant correlations were found between the AR spatial task and other tasks.”

Yes. Thank you. We have added the sentence removed in revision 1 in the first paragraph of the Correlations section.

<u>Reviewer #1</u>:

The authors addressed all my comments from the previous revision. The paper has been improved, and the main issues are now solved. In particular, I appreciated the new figures that now better explain the setup, the structure of the application and how it has been used for the experiments. It is now clear that the use of the Tango framework is not a limitation, since it is clear that the approach can be extended to other frameworks, like ARKit or ARCore (though they are very different in the used techniques, and thus in performance).

Thank you

I still have one comment about the quality of the plots: I see them at a very low resolution in both visualizations. This is especially evident in plots where I cannot read labels very well.

Figures 8 and 9 have been generated again with the required resolution.
